# Hypnosis for the Management of Anticipatory Nausea and Vomiting

**DOI:** 10.6004/jadpro.2015.6.3.4

**Published:** 2015-05-01

**Authors:** Kathy G. Kravits

**Affiliations:** City of Hope, Duarte, California

## Abstract

**CASE STUDY**BJ is a 34-year-old woman who was diagnosed with metastatic breast cancer. She was treated with surgical removal of the primary tumor and sentinel node biopsy. Following surgery, she received chemotherapy. She was given antiemetic therapy prior to and immediately following chemotherapy. She began to experience significant and persistent nausea with intermittent episodes of vomiting after the second cycle of chemotherapy. She completed her chemotherapy but still experienced nausea and vomiting in response to several cues, such as smelling food cooking and going to the hospital. Her nausea and vomiting resulted in segregation from her family during meal time, which negatively impacted her quality of life.

A hypnosis consultation was requested, and BJ was cooperative. She reported feeling very nauseated at the time of the interview. Hypnosis was discussed; her questions were answered, and the potential risks and benefits of hypnosis were reviewed. She agreed that she would like to try hypnosis. A hypnosis assessment was conducted and revealed that she had a history of profound motion sickness and severe, chronic childhood trauma associated with feelings of anxiety and hypervigilance.

The therapeutic suggestions that were used with BJ included hypnotic suggestions for relaxation and removal of discomfort. A metaphor describing the central processing of the anticipatory nausea and vomiting as a thermostat that could be adjusted to reduce and eliminate the sensation was used to suggest that she could control her perceptions and in turn control the nausea. Posthypnotic suggestions included that at the earliest awareness of discomfort, rubbing the throat would eliminate that discomfort, and cooking aromas would be transformed into her favorite fragrance. Reversal went smoothly, and BJ reported satisfaction with the experience.

BJ experienced significant reduction in symptoms after the first session. She had two more sessions, at which time she was able to eat with her family and go to the clinic without discomfort. She was provided a CD with a recording of her hypnosis script to reinforce the face-to-face intervention. She continues to be symptom-free 3 months after treatment with hypnosis.

Persistent nausea and vomiting, which occurs in 10% to 25% of patients receiving chemotherapy, creates a significant burden for patients, increases costs for the health-care system (an average daily treatment cost of $1,854.70) and increases the potential for abandonment of treatment due to the suffering associated with anticipatory nausea and vomiting (ANV; Montgomery, Schnur, & Kravits, 2013; Thompson & O’Bryant, 2013; Roila et al., 2010). The addition of hypnosis to an antiemetic regimen can significantly reduce the potential for the development of ANV, thereby protecting patient quality of life, enhancing the probability of successful disease management, and reducing treatment costs (Kamen et al., 2014; Marchioro et al., 2000; Montgomery, Schnur, & Kravits, 2013; Roscoe et al., 2011).

## BACKGROUND AND LITERATURE REVIEW

Current evidence supports the theory that ANV occurs as the result of classical conditioning paired with patient expectations that nausea and vomiting will occur. It may become persistent and is difficult to treat with medication alone (Kamen et al., 2014).

Classical conditioning is a cognitive process that allows a response generated by exposure to a specific stimulus to occur inappropriately to exposure to a secondary stimulus that would not normally produce such a response (Figure; Kamen et al., 2014). The secondary stimuli (also known as triggers) can precipitate episodes of ANV independently of the original stimulus and may be varied and unique to the experience of the patient. For example, a patient is receiving doxorubicin, a red-colored infusion of chemotherapy. The patient experiences nausea and vomiting after the infusion. The nausea and vomiting persists and is triggered whenever the patient sees the color red outside of the experience of chemotherapy.

**Figure 1:**
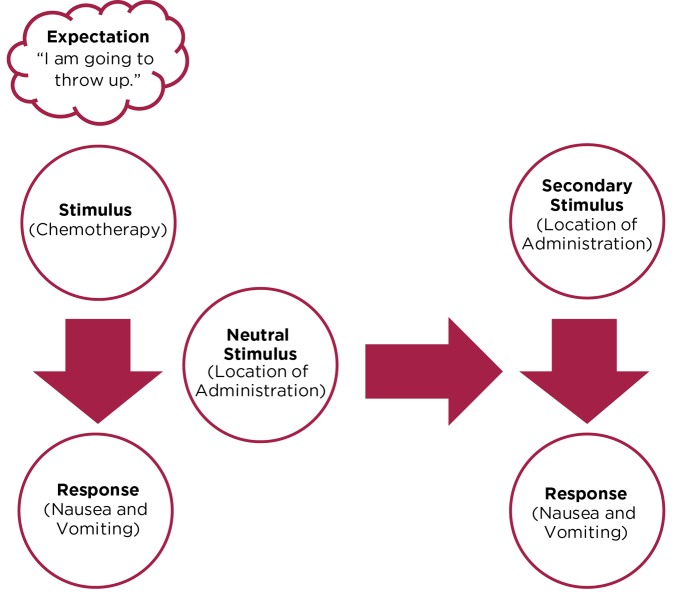
Classical conditioning with expectation.

Another factor that has been identified as contributing to the development of ANV in association with classical conditioning is expectation (Kamen et al., 2014). An expectation is a belief in something or that something will occur. A patient’s closely held belief (the expectation) that he or she will experience nausea and vomiting significantly increases the risk that nausea and vomiting will occur (Kamen et al., 2014). See Table 1 for risk factors associated with the development of anticipatory nausea and vomiting.

**Table 1:**
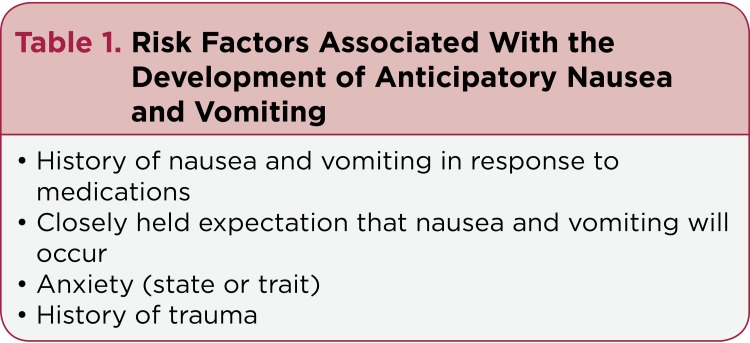
Risk Factors Associated With the Development of Anticipatory Nausea and Vomiting

**Prevention and Management of ANV**

A review of the literature indicates that the most effective way to prevent ANV is to adequately treat patients prior to and immediately following chemotherapy (Kamen et al., 2014; Mustian et al., 2011; Roscoe et al., 2011; Roila et al., 2010). It is important to consider the risk factors for the development of nausea and vomiting when determining an antiemetic regimen (Table 2). The emetogenic potential of the chemotherapy, age, gender, history of nausea and vomiting, susceptibility to motion sickness, anxiety, and expectations of developing nausea and vomiting should be considered (Hesketh, 2008; Roscoe et al., 2011). Research indicates that ANV can be managed most effectively by the use of a combination of medications and psychological techniques such as hypnosis (Hesketh, 2008; Kamen et al., 2014; Mustian et al., 2011; Roila et al., 2010; Roscoe et al., 2011).

**Table 2:**
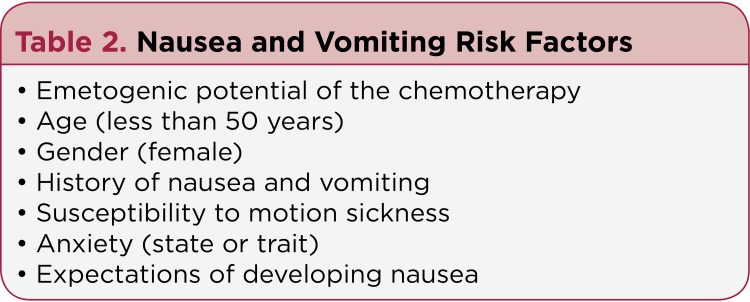
Nausea and Vomiting Risk Factors

**Hypnosis**

One of the first techniques used to control ANV, hypnosis has been found to be safe and efficacious (Kamen et al., 2014; Montgomery, Schnur, & Kravits, 2013; Mustian et al., 2011; Richardson et al., 2007; Roila et al., 2010; Roscoe et al., 2011; Schiff & Ben-Arye, 2011; Thompson & O’Bryant, 2013). Hypnosis is a psychotherapeutic technique practiced between a patient and a trained clinician who uses therapeutic suggestions to produce changes in perception, cognition, affect, mood, behavior, and sensation that are deemed desirable by both parties (Montgomery, Schnur, & Kravits, 2013).

Two of the neural correlates associated with hypnosis are the dorsal left prefrontal cortex (DLPFC), an area of executive control, and the anterior cingulate cortex (ACC), a structure within the salience network. Other structures within the salience network include the anterior insula, the amygdala, and the ventral striatum (Spiegel, 2013; Taylor et al., 2010). Hypnotic modulation of perception uses the somatosensory system as well as the DLPFC and ACC to reset perceptions of symptom intensity (Spiegel, 2013).

Cognitive processes that contribute to the effect of hypnosis are absorption (focused attention), dissociation (relegation of competing stimuli to the edge of awareness), and suggestibility (the willingness to go along with what is being suggested; Spiegel, 2013). Whether or not there is a unique state produced by the hypnotic interaction is a matter of some debate. Suffice it to say that the absorption achieved during the hypnotic experience in conjunction with dissociation, suggestibility, and the patient’s expectation of success creates an opportunity for therapeutic change to occur (Spiegel, 2013; Montgomery, Schnur, & Kravits, 2013). Hypnosis is not well integrated into palliative care due to several factors: myths and misconceptions, lack of sufficient understanding of its mechanism of action, and lack of appropriately trained individuals to provide it (Desai, Chaturvedi, & Ramachandra, 2011; Mottern, 2010; Vandenberg, 2010).

## USE OF HYPNOSIS IN ANV

The safety and efficacy of hypnosis are well established (National Institutes of Health, 1996; Deng et al., 2009; Montgomery et al., 2007; Montgomery, Schnur, & Kravits, 2013). There is also evidence to support its use in the management of ANV (Marchioro et al., 2000; Richardson et al., 2007). As mentioned previously, ANV is a learned response that occurs as the result of classical conditioning and expectations that nausea and vomiting will occur.

Medications are generally ineffective at interrupting the conditioned response and frequently associated with undesirable side effects. Anxiolytics, such as the benzodiazepines, may be useful in reducing the anxiety that often supports the maladaptive conditioned response, but they do not alter the response itself. They are also associated with significant side effects (Thompson & O’Bryant, 2013). Hypnotic interventions constructed to incorporate suggestions that promote desensitization to the stimuli that trigger the nausea and vomiting and that provide alternative responses (relaxation) to the stimuli are effective at eliminating ANV without any associated negative consequences or side effects (Hammond, 2010; Marchioro et al., 2000).

**Hypnotic Intervention: Introduction and Assessment**

The intervention is started with an introduction that includes a definition of hypnosis, a description of the hypnotic process, a review of the role of the hypnotist and the patient, and a discussion of the safety and efficacy of the intervention. Many patients have preconceived ideas about hypnosis, including some misconceptions. It is very important to address any such myths or misconceptions. This dialogue provides the foundation for a collaborative therapeutic relationship between the patient and the hypnotist and for a truly informed consent for the hypnotic experience (Montgomery, Schnur, & Kravits, 2013).

An assessment of the patient’s needs, preferences, and vulnerabilities should be conducted to begin the development of a therapeutic hypnotic intervention (Montgomery, Schnur, & Kravits, 2013). A history of the patient’s experience with relaxation, guided imagery, hypnosis, meditation and other mind/body therapies informs the type of imagery and suggestion that the hypnotist will use in the hypnotic intervention. Identifying preferred sensory experiences assists the hypnotist to build an intervention script that capitalizes on the individual’s unique memories of safe and relaxing environments. Evaluation of recent traumatic experiences is often not done, but it is an important aspect of the assessment. Awareness that traumatic material exists in the patient’s memory allows the hypnotist to construct images and language for the hypnotic script that do not act as triggers for unpleasant emotional content.

**Induction and Deepening**

Induction is composed of a series of actions that promote relaxation. These actions commonly include mindful breathing; progressive muscle relaxation; and pleasant, peaceful imagery. Following induction and successful achievement of relaxation by the patient, suggestions for increasing the depth of relaxation are provided. The suggestions may be framed in direct terms, such as "You are becoming more and more relaxed," or metaphorical terms, such as "You are slowly descending a staircase, and with every step you take, feelings of relaxation deepen and grow" (Hammond, 1990, p. 13).

**Therapeutic Suggestions**

Therapeutic suggestions are constructed to achieve a specific clinical goal (Table 3). In the case of ANV, the suggestions are focused on eliminating vomiting, reducing or eliminating feelings of nausea, and/or transforming perceptions of nausea into less-troublesome sensations that are better tolerated (Dillworth & Jensen, 2010).

**Table 3:**
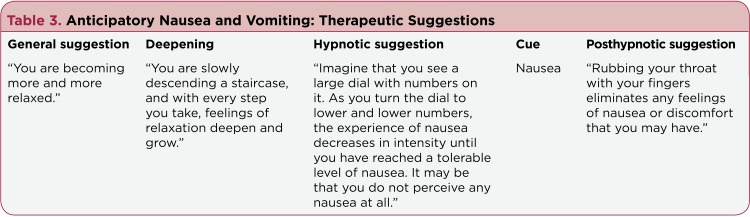
Anticipatory Nausea and Vomiting: Therapeutic Suggestions

Therapeutic suggestions may be constructed to take effect during the hypnotic experience (hypnotic suggestions) or outside of it (posthypnotic suggestions). Posthypnotic suggestions are framed to take effect when a specific cue occurs and often include suggestions for relaxation, transformation of the response to the cue into something acceptable to the patient, and increased feelings of well-being. In some cases, a behavior can be suggested that intensifies the effects of the therapeutic suggestion (e.g., "Rubbing your throat with your fingers eliminates any feelings of nausea or discomfort that you may have"). The creation of suggestions that promote the best possible outcome for that individual includes both hypnotic and posthypnotic elements (Dillworth & Jensen, 2010).

**Reversal**

Reversal is a structured process for terminating the hypnotic experience and returning the patient to his or her usual state of awareness. It includes prompts that reinforce the suggestions for relaxation and feelings of well-being. The suggestion that the eyes will open is often paired with the suggestion that when they open, the patient will be fully alert and aware and no longer in a hypnotized state.

**Summary**

Hypnosis is a valuable option for managing ANV and an even more valuable one for preventing the development of ANV when used before initiation of chemotherapy. The major limitations to its use are the myths and misperceptions held by the public and health-care providers and the lack of trained health-care professionals to provide the service.

Advanced practice nurses can receive training in hypnosis. Two widely respected organizations dedicated to the advancement of the practice of hypnosis are the Society for Clinical and Experimental Hypnosis and the American Society of Clinical Hypnosis. These professional organizations share the highest ethical and educational standards and sponsor hypnosis education. Information regarding educational programs may be found at their websites: www.sceh.us and www.asch.net. Hypnosis is an intervention that should be integrated into our standards of advanced oncology practice.
